# Sitagliptin ameliorates L-arginine-induced acute pancreatitis via modulating inflammatory cytokines expression and combating oxidative stress

**DOI:** 10.3389/fphar.2024.1389670

**Published:** 2024-05-28

**Authors:** Heba M. Eltahir, Hossein M. Elbadawy, Mohannad A. Almikhlafi, Ali M. Alalawi, Ahmed J. Aldhafiri, Yaser M. Alahmadi, Sultan S. Al thagfan, Muayad Albadrani, Saber M Eweda, Mekky M. Abouzied

**Affiliations:** ^1^ Department of Pharmacology and Toxicology (Biochemistry Subdivision), College of Pharmacy, Taibah University, Medina, Saudi Arabia; ^2^ Department of Pharmacology and Toxicology, College of Pharmacy, Taibah University, Medina, Saudi Arabia; ^3^ Department of Clinical and Hospital Pharmacy, College of Pharmacy, Taibah University, Medina, Saudi Arabia; ^4^ Department of Family and Community Medicine, College of Medicine, Taibah University, Medina, Saudi Arabia; ^5^ 5Department of Clinical Laboratory Sciences, College of Applied Medical Sciences, Taibah University, Madinah, Saudi Arabia; ^6^ Biochemistry Department, Faculty of Science, Alexandria University, Alexandria, Egypt; ^7^ Department of Biochemistry, Faculty of Pharmacy, Minia University, Minia, Egypt

**Keywords:** sitagliptin, L-arginine, acute pancreatitis (AP), oxidative stress, inflammatory cytokines

## Abstract

**Background:**

Acute pancreatitis (AP) is an inflammatory condition that resolves spontaneously, but occasionally, develops into systemic inflammation, organ failure and mortality. Oxidative stress and activation of inflammatory pathways represent major players in AP pathogenesis. Current management of AP relies on attenuating injuries to the pancreas and putting the inflammatory process under control. In this study, we investigated the role of sitagliptin in modulating L-arginine-induced AP in rats.

**Methods:**

Swiss rats were subdivided into a healthy control group, AP group (a single dose of L-arginine 250 mg/100 g, intraperitoneal), and sitagliptin + L-arginine-treated group (10 mg sitagliptin/kg body weight/day, orally). Sitagliptin treatment started 1 hour after L-arginine injection and continued for 3days. Biochemical and histopathological investigations were performed on serum and tissue samples collected from test animals.

**Results:**

L-arginine increased pancreatic meyloperoxidase and serum amylase- and lipase activities and serum levels of TNF-α, LT-α, IFN-γ, IL-1α/β, IL-6, IL-10, IL-12, and IL-15. AP animals showed elevated MDA and NO and decreased GSH and serum calcium levels. Histopathological changes were observed by H&E staining. Sitagliptin treatment significantly ameliorated these biochemical and histological changes diminishing the signs of AP.

**Conclusion:**

Sitagliptin treatment was effective in ameliorating L-arginine-induced AP which can be regarded to its anti-inflammatory and antioxidant effect.

## 1 Introduction

Acute pancreatitis is an inflammatory condition that targets the pancreas and could result in an intense systemic inflammatory response leading to multiple-organ dysfunction. Several factors play a role in inducing AP, including gall stones, alcohol abuse, hypertriglyceridemia, and viral infections among others (Jha et al., 2009; Sarshari et al., 2023). Patients with AP show a high mortality rate; however, only symptomatic treatments that aim at supporting the patients are currently utilized, like analgesics, antiemetics, anti-inflammatory drugs and drugs to improve microcirculation (Sandakov et al., 2014).

Despite the fact that the exact mechanisms behind the cause of AP are still controversial, oxidative stress that results from the immense release of reactive oxygen species (ROS) is believed to be the early and central event in AP initiation and prognosis. This can be regarded to the deleterious effect of ROS on macromolecules and their role in activating inflammatory transcriptional factors and hence their damaging effect on pancreatic tissue (Zeybek et al., 2003). It has been also reported that immune response has a major role in the pathogenesis and prognosis of AP both in triggering and regulating these events (Sendler et al., 2013). Damage to pancreatic acinar cells induces leukocyte activation and neutrophils recruitment to the site of inflammation resulting in the release of massive amounts of pro-inflammatory mediators, e.g., TNF and various interleukins. As a result, the balance between the levels of pro-inflammatory and anti-inflammatory molecules is lost in favor of the pro-inflammatory mediators. This promotes the pathogenesis of AP and mediates the systemic inflammatory response (Hegyi et al., 2011). This central role of inflammatory cytokines led to the assumption that an improvement in AP status could be achieved upon regulating the expression of the inflammatory mediators to retain the balance, as several studies reported positive impacts for regulating interleukins (ILs) levels in different inflammatory diseases (Bhatia, 2005; Ni et al., 2013; Scheller et al., 2014).

The L-arginine-induced model of AP represents one of the most commonly utilized animal models to investigate the pathological changes in the pancreas due to its similarity to the human phenotype of the disease (Kui et al., 2014). Its administration induces severe AP, where the severity is dose dependent (Hegyi et al., 2004). The mechanism behind the deteriorative effects of L-arginine on the pancreas is not yet fully elucidated; however, different research groups postulated that L-arginine-induced AP could be attributed to oxidative stress and toxic metabolites, or even metabolic acidosis (Varga et al., 1997; Hegyi et al., 2004; Hardman et al., 2005). In addition, others related its effects to nitric oxide (NO) and inflammatory mediators’ activation (Takacs et al., 1996; Takacs et al., 2002; Rakonczay et al., 2003).

Arginine specifically targets acinar cells in the pancreas without affecting other cellular components of the pancreas (Kui et al., 2015). Its compromising effect on acinar cells induces the release of pro-inflammatory cytokine expressions such as TNF-α and various interleukins (ILs) (Yenicerioglu et al., 2013; Wang et al., 2017).

Dipeptidyl peptidase-4 (DPP-4) is a glycoprotein enzyme found on cell membranes of natural killer (NK) cells, macrophages, B-cells and T-cells among others. It cleaves dipeptides and promotes the breakdown of circulating incretins like glucagon-like peptide-1 (GLP-1) and gastric inhibitory polypeptide, which are responsible for regulating the glucose-dependent insulin release from the pancreas. In addition, DPP-4 possesses other functions that are not related to insulin and glucose metabolism, as it aids extracellular matrix degradation and cytokine production, in addition to stimulating immune responses, and inducing signaling and resistance to anti-mitotic drugs as well (Itou et al., 2013; Shao et al., 2020). Inhibiting DPP-4 via specific inhibitors allows for increasing the availability of incretins (e.g., GLP-1) and gastric inhibitory polypeptide leading to increased insulin release after meals and hence a better regulation of blood glucose levels, which explains the use of such inhibitors for the control of blood glucose levels in diabetic individuals (Zerilli and Pyon, 2007). Sitagliptin is a well-known DPP-4 inhibitor that is used since 2006 to control blood glucose levels in type 2 diabetic patients (Zerilli and Pyon, 2007). However, it has been reported in several studies that sitagliptin, in addition to other DPP-4 inhibitors, exerts additional biological functions unrelated to their blood glucose-lowering effect. The use of DPP-4 inhibitors showed positive effects in cardiovascular disorders, autoimmune diseases, lung disorders and inflammatory conditions via retrieving the endogenous antioxidant capacity that ameliorates oxidative stress-induced damage and modulating immune response (Fan et al., 2003; Eligar and Bain, 2013; Nade et al., 2015; Al-Rasheed et al., 2016; Civantos et al., 2017).

To the best of our knowledge, no studies have utilized sitagliptin in ameliorating L-arginine-induced AP. Based on this; we aimed in this study at investigating the potential role of sitagliptin in ameliorating L-arginine-induced AP in a rat model and the possible mechanisms behind this effect.

## 2 Materials and methods

### 2.1 Materials

Sitagliptin (Merck Sharp & Dohme, Kenilworth, NJ, United States of America) was purchased from local suppliers. Thiobarbituric acid, Dimethoxybenzidine, Ellman’s reagent (5,5′-dithio-bis-(2-nitrobenzoicacid), 1,1′,3,3′-tetramethoxypropane, L-arginine hydrochloride and hexadecyltrimethyl ammonium bromide were obtained from Sigma-Aldrich (St. Louis, MO, United States of America). All other used chemicals of analytical grade were purchased from local suppliers.

### 2.2 Experimental design

Thirty Swiss albino male rats (150–200 g body weight) were utilized in this study. Animals were kept in plastic cages under standard 12 h s light/dark at 18–25°C and were allowed to adapt for 1 week before being involved in the experimental procedures. Rats had free access to a standard diet and water. All experiments with animals were performed in accordance with the guidelines for the care and use of laboratory animals of the National Institutes of Health (NIH publication No. 85–23, revised 1985). The study protocol was approved by members of “The commission of the Ethics of scientific research”, Faculty of Pharmacy, Minia University, Egypt (code number of the project: ES11/2022).

To conduct the experiment, rats were randomly divided into three groups, each of ten rats.

Group І (control group): administered a single intra-peritoneal (IP) dose of normal saline. Starting 1 h after injection, animals received a daily dose of normal saline per oral gavages for three consecutive days.

Group II (AP group): animals received a single dose of L-arginine (250 mg/100 g body weight, freshly prepared, pH 7.0) per IP (Hardman et al., 2005), and received a daily dose of normal saline per oral gavages for three consecutive days.

Group III (sitagliptin-L-arginine group): Animals received a single dose of L-arginine (250 mg/100 g body weight) per IP and after 1 h of L-arginine injection they started to receive a daily dose of 10 mg/kg/day sitagliptin in saline (Januvia^®^, MSD, (Marques et al., 2014)) for three consecutive days per oral gavages.

By the end of the third day, animals were anesthetized using thiopental (50 mg/kg, per IP) and blood was collected by cardiac puncture. After allowing blood to clot for 20–30 min, serum was separated by centrifugation at 2,000 g for 15 min at 4°C. Pancreas tissues were collected promptly after confirming animal death and divided each into two parts. One part was homogenized in ice-cold 0.15 M KCl to a final concentration of 20% (w/v) and stored in aliquots at −80 C. The other part of each pancreas was fixed in 10% formalin to be further processed for histopathological examination.

### 2.3 Methods

#### 2.3.1 Evaluation of serum amylase and lipase activity

Serum amylase- and lipase activity were assessed using commercially available kits according to previously described methods for serum amylase (Klein and Foreman, 1980) and serum lipase (Lott et al., 1986) (Randox^®^ labs, US).

#### 2.3.2 Determination of serum calcium level

Serum calcium concentrations were evaluated colorimetrically by o-cresolphthaline method (Zerwekh and Nicar, 1984) using commercially available kits (Randox^®^ labs, US) according to the manufacturer’s instructions.

#### 2.3.3 Assessment of oxidative stress condition of the pancreatic tissues

Pancreatic content of reduced glutathione (GSH, ab239727, Abcam), malondialdehyde (MDA, ab118970, Abcam), nitric oxide (NO, ab65328, Abcam) and myeloperoxidase (MPO, ab105136, Abcam) activity were assessed using commercially available kits based on the previously published methods and according to the instructions of the manufacturers (Ellman, 1959; Manktelow and Meyer, 1986; Uchiyama et al., 1987; Miranda et al., 2001).

#### 2.3.4 Determination of serum cytokines

Serum levels of a panel of nine different cytokines including IL-1α, IL-1β, IL-6, IL-10, IL-12, IL-15, TNF-α, LT-α and INF-γ were assessed using commercially available ELISA kits according to the manufacturer’s instructions (Aviva Systems Biology, United States of America).

#### 2.3.5 Histopathological examination

Fixed pancreatic tissues were sequentially dehydrated in increasing alcohol concentrations and cleared before being embedded in paraffin blocks. Five µm thick sections were cut and stained with hematoxylin and eosin, or immune-stained against TNF-α or IL-17 using specific polyclonal IgG as described elsewhere (ab220210 and ab214588 respectively, Abcam) (Jammal et al., 2015), after being deparaffinized and rehydrated in a series of decreasing alcohol concentrations. The tissue sections were blindly examined and evaluated under microscope via a specialized histopathologist. Pathological changes were quantified based on the scoring system of Gibson-Corley and his work team (Gibson-Corley et al., 2013). For the quantification, three independent slides were utilized for each test group, from each, five different microscopic fields were investigated.

#### 2.3.6 Statistical analysis

Statistical analysis was performed using GraphPad Prism 6.0 software (GraphPad, San Diego, Ca, United States of America). Data were represented as mean ± SE. Multiple comparisons were carried out using one way ANOVA test followed by Bonferroni test as a multiple comparison post ANOVA test. *p* < 0.05 was selected to indicate statistical significance.

## 3 Results

### 3.1 Serum amylase and lipase activity

**FIGURE 1:**
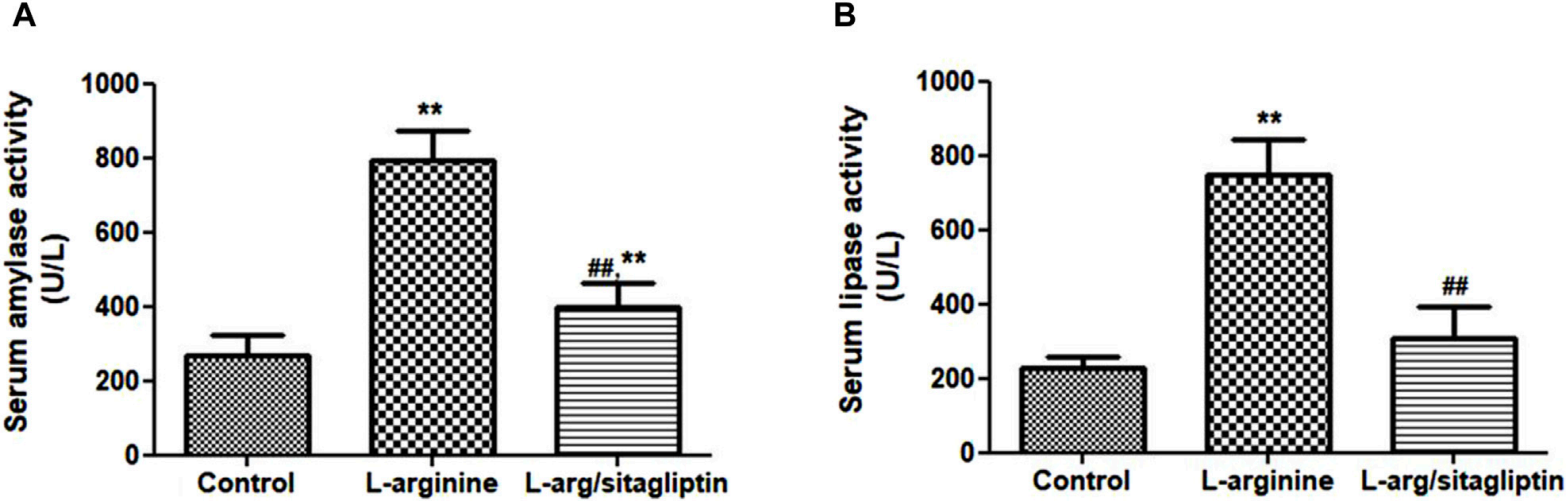
Effect of L-arginine and sitagliptin on serum amylase **(A)** and -lipase **(B)** activity. L-arginine treatment induced a significant increase in serum amylase and lippase activity compared to healthy control. Sitagliptin treatment of pancreatitis animals significantly ameliorated activity of both enzymes compared to animals treated with L-arginine alone.Results are expressed as mean ± SE.* significant difference from control, # significant difference from L-arginine, n = 10. *, #: *p* < 0.05; **, ##:*p* < 0.001. Significance was calculated by ANOVA followed by Bonferroni test as a multiple comparison post ANOVA test.

Three days after induction of AP via IP injection of L-arginine, a significant increase in serum amylase and lipase activities was observed in L-arginine-treated animals compared to healthy control ones. Interestingly, treatment with sitagliptin after induction of AP significantly suppressed the activity of both amylase and lipase in comparison to the AP group (Figures 1A, B, *p* < 0.001). It is to be noted that sitagliptin effectively normalized the activity of lipase to values comparable to the control group (*p* > 0.05), but the amylase activity showed a significant difference when comparing both groups (sitagliptin vs. control, *p* < 0.05).

### 3.2 Effect of sitagliptin on serum calcium level

**FIGURE 2:**
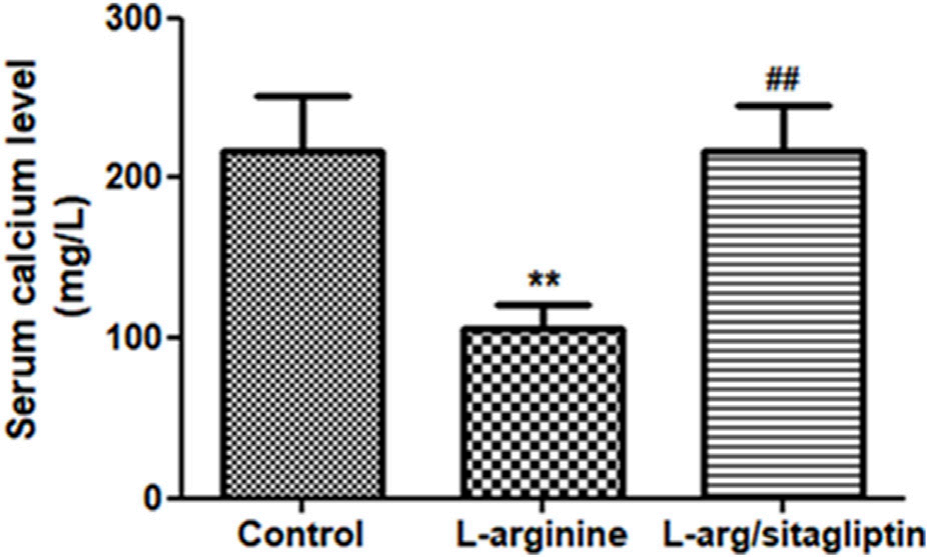
Effect of L-arginine and sitagliptin on serum calcium levels. L-arginine treatment induced a significant depletion of serum calcium compared to healthy control. Sitagliptin treatment of pancreatitis animals significantly retrieved calcium levels compared to animals treated with L-arginine alone. Results are expressed as mean ± SE.* significant difference from control, # significant difference from L-arginine, n = 10 at *, #: *p* < 0.05; **, ##:*p* < 0.001. Significance was calculated by ANOVA followed by Bonferroni test as a multiple comparison post ANOVA test.

L-arginine treatment resulted in alteration of serum calcium levels in the corresponding animals. One can observe a significant reduction of serum calcium level in L-arginine-treated animals when compared to healthy control (*p* < 0.001). In contrast, Sitagliptin treatment successfully retrieved calcium levels in comparison to the AP group (Figure 2, *p* < 0.001). Serum calcium levels showed no significant difference when comparing sitagliptin-treated animals to control ones (*p* > 0.05).

### 3.3 Effect of sitagliptin on pancreatic MPO activity

**FIGURE 3:**
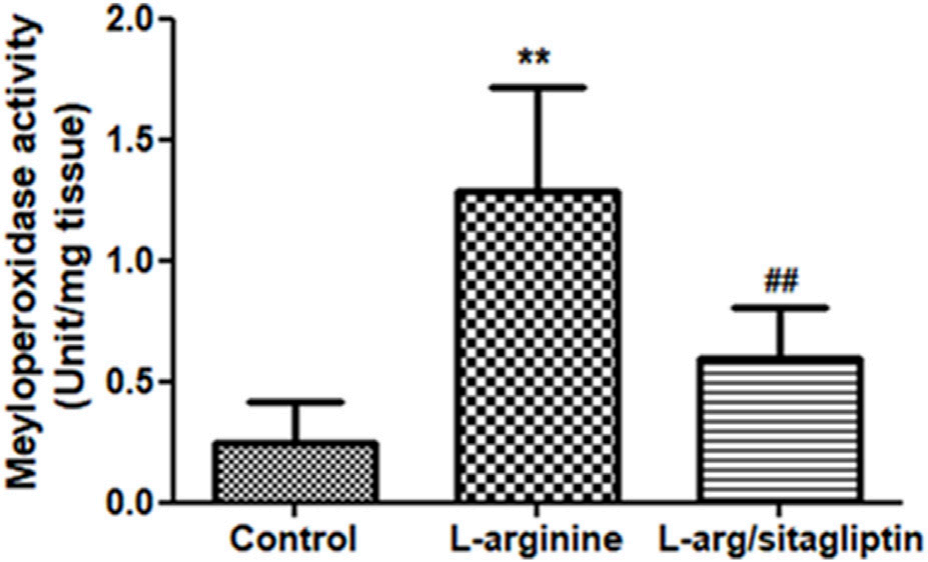
Effect of L-arginine and sitagliptin on serum levels of pancreatic MPO. L-arginine treatment induced a significant increase in MPO in the pancreatic tissue compared to healthy control. Sitagliptin treatment significantly ameliorated MPO activity compared to animals treated with L-arginine alone. Results are expressed as mean ± SE.* significant difference from control, # significant difference from L-arginine, n = 10 at *, #: *p* < 0.05; **, ##:*p* < 0.001. Significance was calculated by ANOVA followed by Bonferroni test as a multiple comparison post ANOVA test.

Upregulation of MPO activity correlates with AP development as reported for different experimental models of the disease (Seyhun et al., 2011). In our study, administration of L-arginine induced a significant increase in pancreatic MPO activity compared to healthy control animals (*p* < 0.001). Notably, administration of sitagliptin after induction of AP resulted in significantly lower MPO activity in the corresponding animals compared to AP animals that did not receive sitagliptin (Figure 3). MPO activity in sitagliptin-treated animals was comparable to the activity in control group animals (*p* = 0.08).

### 3.4 Effect of sitagliptin on pancreatic oxidative stress parameters

L-arginine administration significantly depleted GSH content in the AP group compared to healthy control rats. In the same time, AP group animals showed a significant increase in lipid peroxidation detected in the form of elevated MDA in addition to a significant increase in pancreatic NO content compared to the healthy control group (*p* < 0.05). Interestingly, administration of sitagliptin after induction of AP significantly replenished GSH content, ameliorated lipid peroxidation and suppressed the increase in pancreatic content of NO in treated animals compared to those who did not receive sitagliptin after AP induction, however, the values of this treated group remained significantly higher than healthy control (Table 1).

**TABLE 1:** Effect of L-arginine and sitagliptin on pancreatic content of GSH, MDA and NO activity.

Parameters	Control	L-arginine	L-arg/sitagliptin
GSH (µmol/g tissue)	2.822 ± 0.15	1.026 ± 0.05^*^	1.549 ± 0.087^*,#^
MDA (nmol/g tissue)	17.042 ± 1.013	36.899 ± 1.2^*^	26.65 ± 1.14^*,#^
NO (µmol/g tissue)	2.194 ± 0.229	5.66 ± 0.233^*^	3.725 ± 0.23^*,#^

Results are expressed as mean ± SE. *: significant difference compared to control, #: significant difference compared to L-arginine, (n = 10) at *p* < 0.05. Significance was calculated by ANOVA, followed by Bonferroni test as a multiple comparison post ANOVA, test.

### 3.5 Effect of sitagliptin on serum cytokines

**FIGURE 4:**
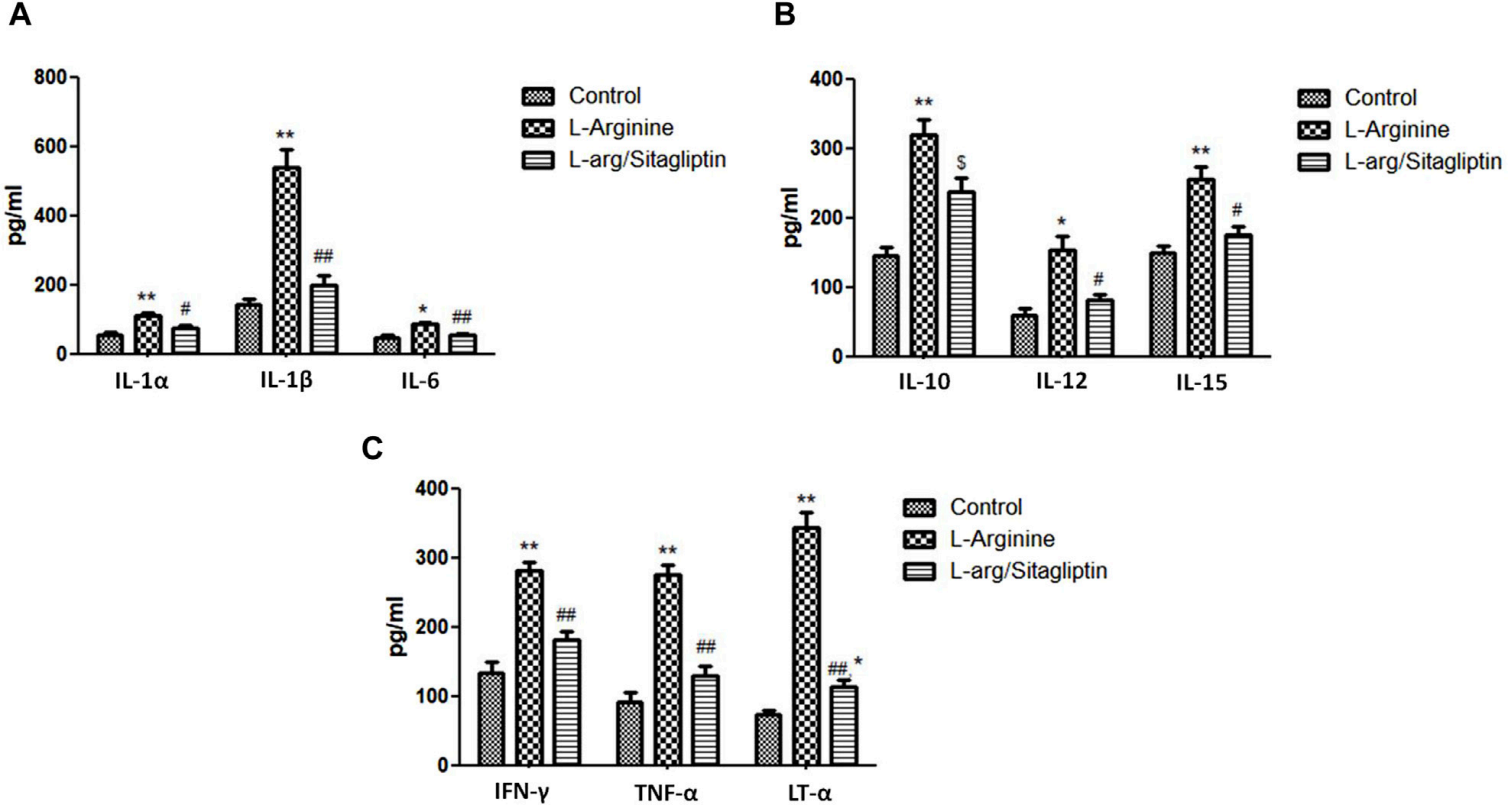
Effect of L-arginine and sitagliptin on the level of circulating cytokines. L-arginine treatment induced a significant increase in serum cytokines compared to healthy control. Sitagliptin treatment of pancreatitis animals significantly ameliorated cytokines levels compared to animals treated with L-arginine alone.Results are expressed as mean ± SE.* significant difference from control, # significant difference from L-arginine, n = 10 at */#: *p* < 0.05; **/##: *p* < 0.001. Significance was calculated by ANOVA followed by Bonferroni test as a multiple comparison post ANOVA test.

When compared to healthy control animals, treatment with L-arginine induced a significant increase in the serum levels of different cytokines including IL-1α, IL-1β, IL-6, IL-10, IL-12 and IL-15 in addition to TNF-α, Lymphotoxin-α (LT-α) and IFN-γ (*p* < 0.05). Sitagliptin administration effectively and significantly reduced the level of the mentioned cytokines (*p* < 0.05) in the corresponding animals in comparison to L-arginine treated animals with the exception of IL-10, where the sitagliptin treatment reduced its level but not to a significant level compared to AP group (*p* = 0.051) (Figures 4A-C). It is to be noted that the level of the investigated cytokines were not significantly different in sitagliptin-treated animals and control group animals (*p* > 0.05) except for IL-10 and LT-α, which levels were significantly higher in sitagliptin treated animals than the control (*p* = 0.014 and *p* = 0.038 respectively).

### 3.6 Histological examination

#### 3.6.1 Histopathological evaluation with H&E staining

**FIGURE 5:**
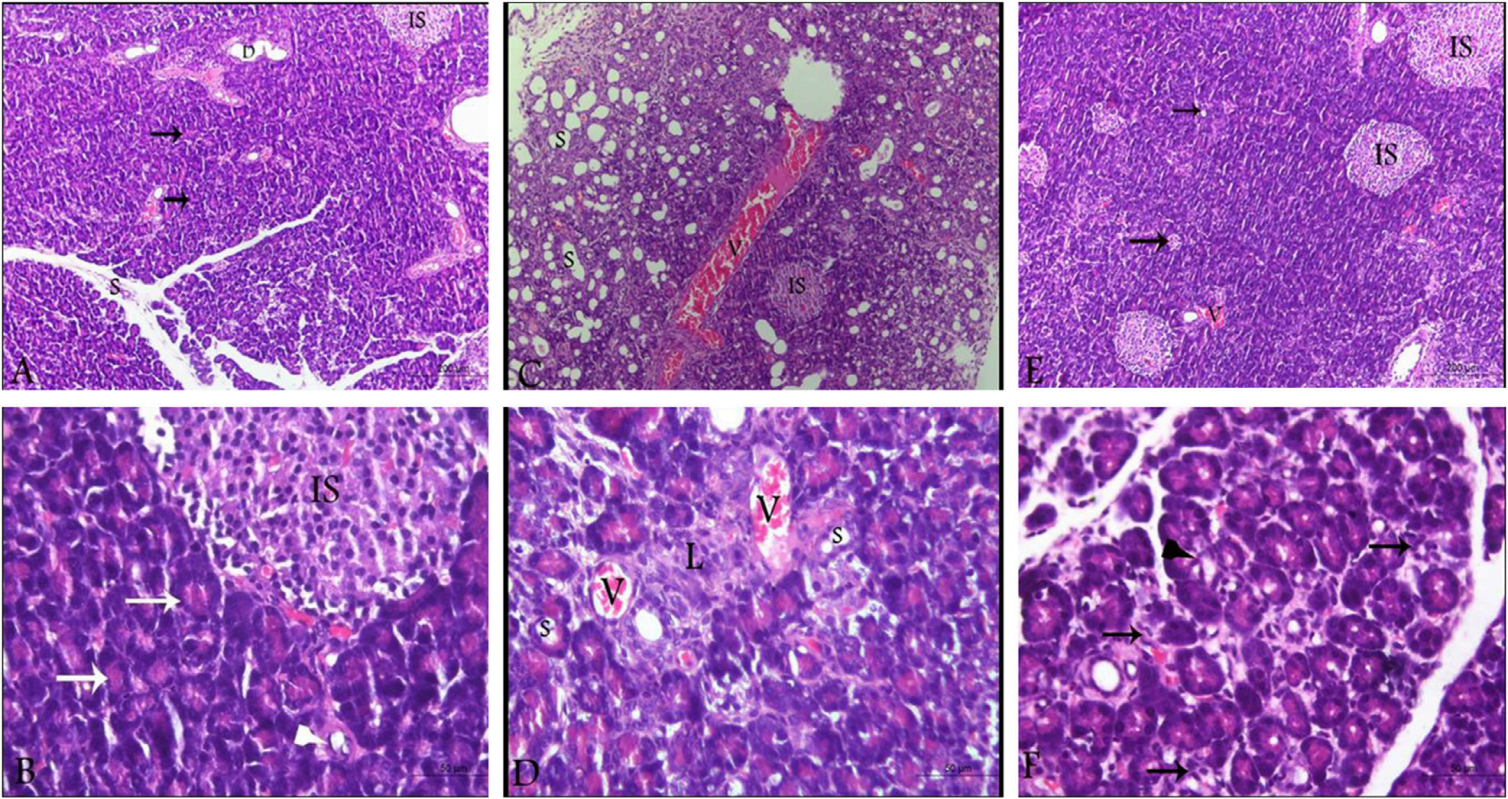
Photomicrographs of pancreatic sections from the different test groups (hematoxylin and eosin). **(A,B)** Pancreatic sections from control animals, showing normal histological architecture of pancreatic lobules. Cells of the islets of Langerhans cells, the acini and the ductal system look normal as well with active secretory acini (white arrow). **(C,D)** sections from L-arginine-treated animal reflecting inflamed pancreatic lobules, thickened septa as well as severely congested blood vessels and inactive degenerating acini (S) and Islets of Langerhans (IS). **(E,F)** sections from sitagliptin-treated animals after induction of AP showing normal lobules with thin septa and few congested blood vessels. Acini and Islets of Langerhans are mostly normal with cystic dilatation (arrows, E; arrowhead, **(F)** and infiltrated lymphocytes (arrows, **(F)**. Upper panel 100x (scale bars 200 µm), lower panel ×400 magnification (scale bars 50 µm). V: vessels, IS: islets of Langerhans, S: secretory acini.

By investigating pancreatic sections from healthy control animals, no pathological manifestations were observed. The sections showed normal pancreatic lobules with normal islets of Langerhans, active acini (arrows, Figures 5A, B) and prominent ducts (arrowheads, Figure 5B) separated by thin septa of connective tissue as observed in Figures 5A, B with no signs of cystic dilataion, edema, congestion or fibrosis (Table 2). Treating the animals with L-arginine adversely affected the architecture of the pancreatic tissues of the corresponding animals. In this test group, one can detect inflamed pancreatic lobules separated by thick septa of connective tissue that enclose congested blood vessels. Inactive secretory Acini (S) within the lobules show signs of degeneration associated with cystic dilatation. Also, signs of degeneration and blood vessel congestion were observed within pancreatic islets of Langerhans in AP group animals in addition to infiltrated lymphocytes (Figures 5C, D). Using the previously mentioned scoring system, sections from this test group showed signs of cystic dilataion, edema, congestion and fibrosis, as well as increased number of infiltrating lymphocytes and degenerating secretory and endocrine cells (Table 2). Noteworthy, treating AP animals with sitagliptin alleviated most of the deleterious histological changes induced by L-arginine. Sections from the pancreas tissues of this group showed normal pancreatic islets of Langerhan’s and normal pancreatic lobules separated by thin connective tissue septa, however, slightly congested blood vessels were still detectable. Also, acini appear mostly normal and active with few acini that still show signs of degeneration, cystic dilatation (Figures 5E, F) and infiltrated lymphocytes (black arrows, Figure 5F; Table 2).

**TABLE 2:** Histopathological scoring of pancreatic sections from the different test groups.

	Control	L-arginine	L-arg/sitagliptin
Cystic dilatation/field	0	3	0
Edema	0	3	1
Congestion	0	4	1
Fibrosis	0	4	1
Degenerated secretory cells/field	0	132 ± 2.821*	14 ± 0.88^#^
Degenerated endocrine cells/field	0	18 ± 0.11*	6 ± 0.033^#^
No. of infiltrated lymphocytes/field	4 ± 0.08	26 ± 0.2*	8 ± 0.02^#^

Scoring was achieved based on Gibsons-Corley’s scoring system (Gibson-Corley et al., 2013), where 0 indicates no pathological change, one indicates <25% pathological changes, two indicates 26%–50%, three indicates 51%–75% and four indicates 76–100 pathological changes.

Data were extracted from three independent slides per group, using five, different microscopic fields/slide. Results are expressed as Mean ± SE. *: significant compared to control, #: significant compared to L-arginine. *p* values less than 0.05 indicate significantly different results.

#### 3.6.2 Effect of sitagliptin on pancreatic TNF-α and IL-17 expression

**FIGURE 6:**
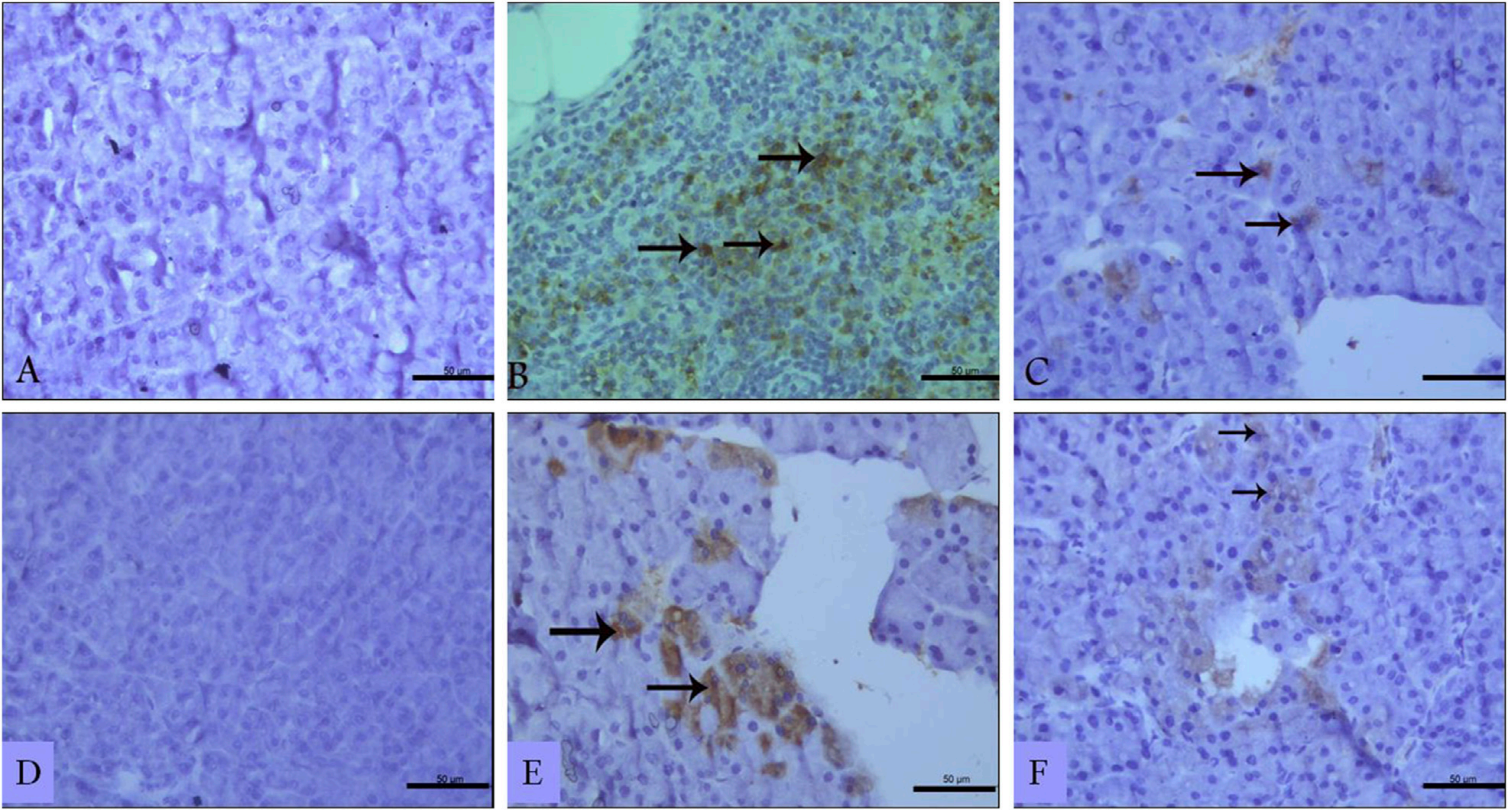
Effect of L-arginine and sitagliptin on TNF-α **(A–C)** and IL-17 **(D–F)** expression in pancreatic sections from the different test groups. Sections from the L-arginine group show a positive expression signals for both cytokines. A much weaker signal can be detected in sections from sitagliptin-treated group. Scale bar: 50 µm.

Immunohistochemistry of pancreatic sections three days after L-arginine administration reflected positive expression signals for both TNF-α (Figure 6B) and IL-17 (Figure 6E) in pancreatic tissues in the AP group compared to healthy control animals that showed almost no staining signal (Figures 6A, D respectively). Sitagliptin treatment after induction of AP resulted in reducing pancreatic tissue expression of TNF-α and IL-17 compared to L-arginine treated group (Figures 6C, F). Quantification of the expression signal intensity in the different test groups reflected a significant increase in TNF-α and IL-17 in AP group compared to healthy control, whereas sitagliptin treatment resulted in significantly lower expression of both mediators compared to AP group that didn’t receive further treatments (Table 3, *p* < 0.05).

**TABLE 3:** Quantification of TNF-α and IL-17 expression.

	Control	L-arginine	L-arg/sitagliptin
TNF-α	59.87 ± 0.04	226.976 ± 1.16*	106.045 ± 2.08^#^
IL-17	52.013 ± 0.08	211.005 ± 2.24*	107.718 ± 1.87^#^

Expression was quantified based on immunostaining signal intensity using ImageJ software. Data were extracted from three independent slides per group, using five, different microscopic fields/slide. Results are expressed as Mean ± SE. *: significant compared to control, #:significant compared to L-arginine. *p* values less than 0.05 indicate significantly different results.

## 4 Discussion

AP represents a self-limited, emergency condition that can be induced by different triggers, however, it can easily develop into a life-threatening systemic inflammatory condition with multiple organ failures leading to high rates of morbidity and mortality (Whitcomb, 2006). Reactive oxygen species (ROS) have been shown to play a major role in AP by altering the nature of zymogen granules’ membranes, resulting in leakage of digestive enzymes, e.g., amylase, and lipase, and cellular proteins within acinar cells and pancreatic tissue, with the subsequent auto-digestion. This event is accompanied by the early activation of inflammatory pathways via activation of nuclear factor-κB (NF-κB) and its downstream inflammatory mediators (e.g., tumor necrosis factor- α (TNF-α) and IL-6), which represent a main event in AP pathogenesis (Ohmuraya and Yamamura, 2008). These molecular changes were reported in l-arginine-induced AP model, which is one of the most commonly used AP models recently (Hegyi et al., 2004).

These mediators attract immune cells such as macrophages and neutrophils, and induce their infiltration and activation in the pancreas, representing another fundamental event in the evolution of AP (Yu et al., 2002).

Activated macrophages are either M1-type, producing pro-inflammatory mediators (e.g., IL-1β, IL-6, IL-8, IL-12, and TNF-α), or M2-type, producing anti-inflammatory molecules such as IL-4, IL-10, TGF-β, and tissue repair factors (Van Dyken and Locksley, 2013; Zhang et al., 2020). Whereas M1 macrophages propagate the inflammatory response, M2 cells ameliorate inflammation via interrupting downstream signaling pathways of pro-inflammatory mediators allowing better control of the inflammatory response in AP (Virlos et al., 2004).

In the current study, we aimed at exploring the potential effect of the DPP-4 inhibitor sitagliptin on L-arginine-induced AP in a rat animal model. In agreement with previous reports, L-arginine injection induced oxidative stress as observed by the upregulation of different oxidative stress markers including MDA and NO, and depletion of GSH. It also increased MPO-, lipase- and amylase activity and resulted in depletion of serum calcium as well (Condon et al., 1975). An overall increase in various ILs including IL-1α/IL-1β, IL-6, IL-10, IL-12 and IL-15 was observed together with an increase in the level of circulating IFN-γ, TNF-α and LT-α. Histological investigation revealed positive expression signals for the pro-inflammatory cytokines TNF-α and IL-17 in pancreatic tissue sections after induction of AP.

Interestingly, sitagliptin administration reduced MPO-, lipase- and amylase activity. It also normalized calcium levels and ameliorated the signs of L-arginine-induced oxidative stress as it retrieved antioxidant capacity and replenished GSH content which represents a part of the pancreas endogenous antioxidant capacity. It also resulted in a significant reduction in serum levels of pro-inflammatory ILs, IFN-γ, LT-α and TNF-α. In addition, sitagliptin treatment caused a downregulation of IL-17 and TNF-α expression signals in pancreatic sections as observed by histopathological investigation.

In response to increased lipase activity upon autodigestion of pancreatic cells, free fatty acids (FAAs) are liberated from visceral fat into the circulation. These FFAs deplete serum calcium by forming Ca+2-FFA insoluble complexes, resulting in a characteristic reduction of total serum calcium level as observed by us and others (Ahmed et al., 2016).

Tissues utilize endogenous GSH, catalase, superoxide dismutase (SOD), thioredoxin and thiol-proteins to evade the deleterious effects of ROS. As mentioned before, L-arginine induces oxidative stress via superoxide, NO and peroxynitril radicals, which easily depletes the endogenous antioxidant reserves, as observed in the current study and reported by others (Zhang et al., 2010). In addition, the acute inflammatory reaction induced by L-arginine induces Neutrophils to be recruited and activated within the inflamed pancreas resulting in upregulation of pancreatic MPO activity; an enzyme that possesses potent pro-inflammatory properties and can be used as a valuable diagnostic factor for AP in animal models and patients as well (Rayner et al., 2014).

One of the hallmarks f acute pancreatitis is the increased levels of the proinflammatory cytokine IL-6, which is released by macrophages that become activated within the inflamed pancreas. Consequently, IL-6 induces the release of IL-17, IFN-γ and IL-12 from differentiated T-cells, and the release of IL-1β, IL-6 and TNF-α from infiltrating macrophages under the control of NFκB (Watanabe et al., 2017).

It is to be noted that IL-17, IL-12 and IL-15 areall pro-inflammatory cytokines that are upregulated in response to L-arginine-induced oxidative stress in AP and play major roles in inducing the production and release of multiple cytokines. Within the inflamed pancreas, IL-17 is produced mainly by T helper-17 cells (Th-17) and injured acinar cells in addition to other cells (Ni et al., 2013), inducing the release of TNF-α, IL-1β, IL-6 and IL-8 (Ni et al., 2013).

On the other hand, IL-12 and IL-15 induce transcription and release of IFN-γ by active NK cells (de Weerd and Nguyen, 2012). Generally, IFN-γ released by NK cells has multiple physiological roles, in addition to its role in tumor genesis and inflammation as it stimulates the release of variable pro-inflammatory cytokines (de Weerd and Nguyen, 2012). Reports have shown that IFN-γ serum levels are upregulated in patients with AP (Bhatnagar et al., 2003).

Notably, multiple inflammatory mediators, i.e., IL-17, IL-6 and IL-10 as well as IL-12 and IL-15 have been shown to play a main role in the pathogenesis of pancreatitis, and their serum levels have been reported to correlate with disease severity in AP patients (Pezzilli et al., 1999; Kamei et al., 2010; Li et al., 2021).

Both TNF-α and TNF-β (recently known as Lymphotoxin-α, LT-α) are inflammatory mediators belonging to the same family, TNF superfamily and become upregulated in pancreatitis. TNF-α is involved as a main player in the pathogenesis of AP and becomes upregulated during the AP-induced inflammatory response, however, its serum level doesn’t usually correlate with the severity of the inflammation in AP due to its prompt hepatic clearance (Grewal et al., 1994; Malleo et al., 2007). LT-αwhich is produced mostly by T-cells, CD+8 cells and NK cells, is linked to cell differentiation and apoptosis as well as various diseases including autoimmune pancreatitis (Seleznik et al., 2012).

As mentioned before, recruited macrophages in AP release IL-1β, which acts as a chemotactic factor allowing neutrophil infiltration into the pancreas with the consequent release of further chemokines and pro-inflammatory cytokines leading to aggravating the inflammatory response (Garlanda et al., 2013). Both IL-1α and IL-1β are inflammatory mediators that are shown to be upregulated in AP and bind the same receptor, resulting in activation of downstream NFκB signaling followed by transcription of its downstream pro-inflammatory cytokines, including IL-6 and TNF-α. It is to be mentioned that IL-1α is over-expressed in response to oxidative stress and pro-inflammatory cytokines, acting as a danger signal upon cell necrotic injury in several pathological conditions including AP (Fink and Norman, 1997; Dinarello, 2011; Malik and Kanneganti, 2018).

In contrast to the aforementioned cytokines, IL-10 is an anti-inflammatory cytokine that is produced by M2 macrophages, T-cells, B-cells and dendritic cells to combat the production of inflammatory mediators via inhibiting NFκB activation, and aid the regeneration phase of the pancreas after AP (Mahony et al., 2016). In agreement with the data presented in the current study, serum levels of IL-10, along with IL-1 and IL-6 have been previously shown to be associated with severe AP (Vasseur et al., 2014). In fact, IL-10 can be released from T-cells in response to IL-6, modulating the inflammatory and the immune responses (Ouyang and O'Garra, 2019). This pivotal role of IL-10 enables it to be a main player in inflammatory conditions, as it becomes over-expressed and released by the tissues involved in the inflammatory response in an attempt to circumvent the propagation of the inflammatory response (Murray, 2006). It has been reported to counteract the release of the pro-inflammatory cytokines IL-1α/β, IL-6, IL-12 and TNF-α from various immune cells, indicating its role in resolving the inflammatory status by manipulating the immune response (Ouyang and O'Garra, 2019).

Sitagliptin is a DPP-4 inhibitor that is used as an anti-diabetic drug and was shown to exert antioxidant and anti-inflammatory effects in different pathological conditions in kidneys, liver and cardiovascular system (Nade et al., 2015; Al-Rasheed et al., 2016; Civantos et al., 2017). It acts mainly by increasing insulin secretion from the pancreas via inducing the interaction of GLP-1 with its receptor (GLP-1R). This interaction activates a cAMP-mediated signaling pathway which is involved in regulating various activities including inflammatory response and blood pressure (Hou et al., 2016). It has also been reported to suppress macrophages infiltration in murine nephritis model and to interfere with NFκB signaling and hence preventing inflammation, in addition to reducing the levels of circulating TNF-α and other inflammatory mediators (Satoh-Asahara et al., 2013; Higashijima et al., 2015).

These anti-inflammatory effects of sitagliptin resulted in the observed downregulation in serum levels of pro-inflammatory ILs, IFN-γ, LT-α and TNF-α which are mainly released by these cells. Reduced expression of inflammatory mediators adversely affects the activity of NO synthesis, alleviating the oxidative stress and allowing the replenishment of the endogenous antioxidant defense mechanisms. Within the pancreas, the antioxidant effect of sitagliptin allows it to stabilize and protect biological membranes against peroxidation via scavenging excessive ROS. This prevents the premature activation and leakage of digestive enzymes, including lipase, from the pancreatic zymogen granules and hinders auto-digestion and lipolysis and the subsequent hypocalcemia as mentioned before, explaining the observed results regarding these parameters in the current study (Civantos et al., 2017).

Despite the effectiveness of DPP-4 inhibitors as blood glucose-lowering agents with positive impact on the cardiovascular and renal system in diabetic patients, a wide controversy is detected among researchers regarding the effect of this drug family on pancreas integrity. However, more studies are needed to get a conclusive outcome regarding these drugs.

On one side, some Meta analysis studies identified a minimal risk of pancreatic events in diabetic patients chronically treated with DPP-4 inhibitors (Pinto et al., 2018). This drawback of DPP-4 use was in part explained relying on the mechanism of action of these drugs, as they increase insulin secretion and suppresses glucagon secretion, resulting in pathological alterations within the pancreatic tissue and increasing risk of pancreatitis and pancreatic cancer as well (Matveyenko et al., 2009; Andersen et al., 2013), taking into account that these findings were detected after chronic use of DPP-4 inhibitors.

On the other side, other research groups have reported no significant association between the use of DPP-4 inhibitors and pancreatitis, suggesting their safety on the pancreas and the lack of association between their use and pancreatitis (Buse et al., 2017; Tseng et al., 2017; Dicembrini et al., 2020).

As observed here, sitagliptin treatment alleviated AP symptoms on the cellular and molecular level possibly by interrupting signaling pathways that could include but not limited to the earlier identified signaling pathway for sitagliptin, NFκB (Satoh-Asahara et al., 2013; Higashijima et al., 2015), resulting in a global modulation of the inflammatory cytokines’ profile. This proposes that sitagliptin may exert its effect in AP via retrieving the intricate balance between pro-inflammatory and anti-inflammatory mediators.

## Data Availability

The raw data supporting the conclusion of this article will be made available by the authors, without undue reservation.
